# A new species of *Tamarixia* Mercet (Hymenoptera, Eulophidae), parasitoid of *Trioza aguacate* Hollis & Martin (Hemiptera, Triozidae) in Mexico

**DOI:** 10.3897/zookeys.368.6468

**Published:** 2014-01-07

**Authors:** Zoya Yefremova, Graciela González-Santarosa, J. Refugio Lomeli-Flores, Néstor Bautista-Martínez

**Affiliations:** 1 Department of Zoology, The George S. Wise Faculty of Life Sciences, Tel Aviv University, Tel Aviv, 69978, Israel; 2 Posgrado en Fitosanidad, Colegio de Postgraduados, Km 36.5 carretera México-Texcoco, Montecillo, Texcoco, Edo. de México, 56230, México

**Keywords:** Insecta, Chalcidoidea, *Tamarixia aguacatensis*, *Trioza aguacate*, *Persea americana*, Mexico

## Abstract

*Tamarixia aguacatensis* Yefremova, **sp. n.** (Hymenoptera: Eulophidae: Tetrastichinae) is described from Mexico as a parasitoid of the avocado psyllid, *Trioza aguacate* Hollis & Martin (Hemiptera: Triozidae). *Trioza aguacate* is a serious pest of avocado, *Persea americana* Miller. A key to the species of *Tamarixia* Mercet in Mexico is given.

## Introduction

The Mexican fauna of Psyllidae is poorly known, and even less known there are psyllid parasitoids. At least four *Tamarixia* Mercet (Eulophidae: Tetrastichinae) species have been recorded in Mexico as psyllid parasitoids: *Tamarixia leucaenae* Bouček from *Heteropsylla cubana* Crawford (Psyllidae: Ciriacreminae), *Tamarixia triozae* (Burks) from *Bactericera cockerelli* (Sulc) (Psyllidae: Triozinae) (Burks 1943), *Tamarixia radiata* (Waterston) from *Diaphorina citri* Kuwayama (Psyllidae: Diaphorinae) (Waterston 1922), and *Tamarixia schina* Zuparko from *Calophya schini* Tuthill (Psyllidae: Calophyidae) (McClay 1990; Lomeli-Flores and Bueno Partida 2002; Alvarez-Zagoya and Cibrian-Tovar 1999; Zuparko et al. 2011).

The most studied species is *Tamarixia triozae*, which was first recorded by Lomeli-Flores and Bueno Partida (2002) from a collection on tomato crops at Michoacán with a level of parasitism of 20–85% on *Bactericera cockerelli*. This species is common as a *Bactericera cockerelli* parasitoid in field crops of some solanaceous plants such as tomatillo (*Physalis philadelphica* Lam.), tomato (*Solanum lycopersicum* L.), potato (*Solanum tuberosum* L.), eggplant (*Solanum melongena* L.), and peppers (*Capsicum annuum* L.). *Tamarixia radiata* was introduced to Mexico for the biological control program against *Diaphorina citri*; this parasitoid species has a wide distribution in Mexico and is now common in most Mexican citrus-growing areas (González-Hernández et al. 2009). This species is mass-reared for the augmentative biological control by Koppert México. As part of a federal program (Campaña Fitosanitaria de Prioridad Nacional contra el HLB: http://www.senasica.gob.mx/?id=4512) in Mexico there are two facilities designated for *Tamarixia radiata* mass rearing, one in Colima and the other one in Yucatán. In April, 2013, alone more than 465,900 parasitoids were released in the citrus areas in seven Mexican states. Elsewhere, the other two species (*Tamarixia leucaenae* and *Tamarixia schina*) were established as biological control agents of the invasive psyllids *Heteropsylla cubana* in Africa and Asia (Day 1999; Rao et al. 2000) and *Calophya schini* in California (Zuparko et al. 2011). The latter species (*Tamarixia schina*) is now common in Mexico as a parasitoid of *Calophya schini* on peppertrees (*Schinus molle* L.). Apparently, this parasitoid disperseed to Mexico from California on its own, and no further studies on it have been conducted. In addition to these psyllid species as hostsof *Tamarixia*, another species of this genus was recovered from the avocado psyllid, *Trioza aguacate* Hollis & Martin (Hemiptera: Triozidae).

The avocado psyllid was discovered for the first time in Mexico in 1995, on avocado trees (*Persea americana* Miller) (Hollis and Martin 1997). This species was originally reported in Uruapan, Michoacán, and later dispersed in most of the avocado growing areas of that state. This pest affects the native Mexican avocado trees including those of the Hass variety. The main damage is caused by the nymphs. Adults deposit their eggs along the centre ribs of young leaves, and the nymphs establish themselves on the leaf ribs and on the tender stems, causing bud deformities. This species is abundant only in spring, when avocado trees develop most of the new buds. Recently, parasitoids in the host nymphs have been detected. From them a species of *Tamarixia* has been reared which does not correspond to any of the previously described taxa in the genus.

## Material and methods

**Figure 1.**
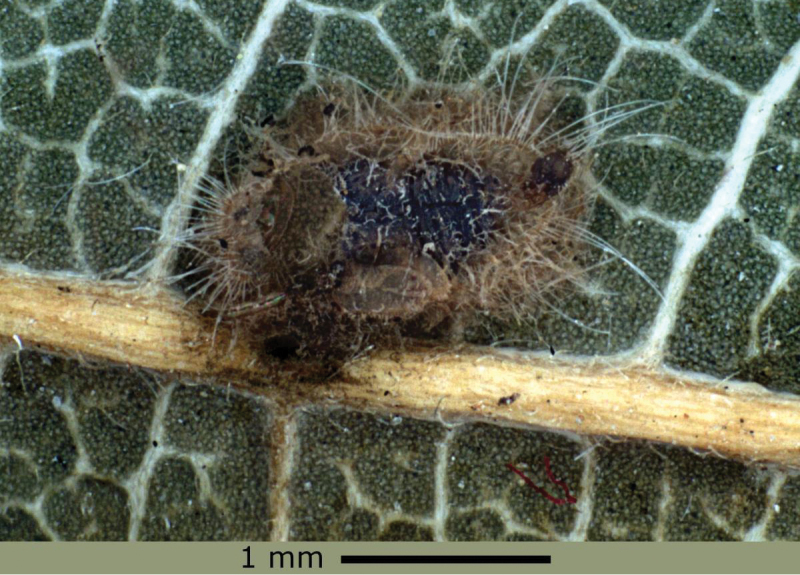
Mummy of *Trioza aguacate* nymph.

Periodic samples were taken in avocado groves in the town of Salvador Escalante, Michoacán, from January 2012 to January 2013; however, presence of the parasitoid was detected only in April and May 2012. To recover some of the parasitoids, buds and avocado leaves with parasitized nymphs of *Trioza aguacate* were collected; these are recognized by their ochre brown tone (Fig. 1). No more than 10 mummies per jar were collected.

The collected material was placed in glass jars covered with organza fabric to wait for the parasitoids to emerge. Overall parasitism of the nymphs was 14.6%; but when we recorded only the large nymphs the percent parasitism was 46.7%.

After the parasitoids were processed, pictures were taken of the diagnostic characteristics to compare this species with the already described species (Graham 1987; LaSalle 1994; Zuparko et al. 2011). The pictures were taken using the Scanning Electron Microscope (JEOL JSM 6390) and a stereomicroscope. Also, a sample of 36 buds was collected on 11 May 2012; the total number of nymphs and the parasitized nymphs were recorded per each 5 cm bud.

Morphological terminology follows that of Graham (1991). F1 – first segment of antennal funicle, F2 – second segment, F3 – third segment, F4 – fourth segment, C1 – first segment of clava, C2 – second segment of clava, C3 – third segment of clava; SMV – submarginal vein, MV – marginal vein, PMV – postmarginal vein, SV – stigmal vein, POL – the minimum distance between the posterior ocelli, OOL – the minimum distance between the eye margin and the adjacent posterior ocellus. Absolute measurements are given in millimetres (mm) for body and fore wing length; for other dimensions, relative measurements are used. Observations and measurements were made using a Nikon dissecting microscope (top magnification of 63 ×) with a 100-division linear scale micrometer.

The following acronyms are used for the depositories of specimens:

CNIN The National Insect Collection at the Instituto de Biología, Universidad Autónoma de Mexico, Mexico City, Mexico.

FSCA Florida State Collection of Arthropods, Gainesville, Florida, USA.

USNM United States National Museum of Natural History, Washington, D.C., USA.

TAUI The National Collection of Insects, Zoological Museum, Department of Zoology, Tel Aviv University, Tel Aviv, Israel.

## Taxonomy

### Genus Tamarixia

Mercet, 1924

http://species-id.net/wiki/Tamarixia

#### Type species.

*Tamarixia bicolor* Mercet, 1924: 57 (original designation).

#### Diagnosis.

*Tamarixia* can be distinguished by the following combination of features: fore wing with a single seta on the dorsal surface of the submarginal vein, propodeum without a Y-shaped carina; plicae and paraspiracular carinae absent, midlobe of mesoscutum with 2 pairs of long adnotaular setae (three pairs setae in *Tamarixia dahlsteni* Zuparko, 2011) and additional 2 pairs of short setae in the upper part in a horizontal row and 1 seta near notauli in *Tamarixia aguacatensis* sp. n. (Fig. 7). The anterior margin of the female hypopygium is almost straight, and the males have exceptionally long genitalia. An additional diagnostic character is that the toruli are closer to eye margin than to each other. Species are generally shiny black, but may have yellow markings on the gaster and/or head. The gaster of the female subcircular to ovate; one seta of each cercus 1.5 times or more the length of the next longest seta.

#### Biology.

Species of *Tamarixia* are primary ectoparasitoids of psyllids (Graham 1987, 1991; Bouček 1988a, 1988b; LaSalle 1994; Brothers and Moran 1969; Moran et al. 1969; Noyes 2013) and parasitize immature stages of *Trioza* (Hemiptera, Psyllidae) (Mead 1994).

#### Distribution.

*Tamarixia* is a cosmopolitan genus, with about 50 described species (Noyes 2013), most of them in Palearctic. Zuparko et al. (2011) listed 47 species of *Tamarixia* in the world but the authors missed 3 species: *Tamarixia krascheninnikovi* (Kostjukov, 1990), *Tamarixia fulvus* Yefremova & Yegorenkova, 2009 and *Tamarixia psyllae* Yefremova & Yegorenkova, 2009 (Kostjukov 1990; Yefremova and Yegorenkova 2009).

#### Identification.

Keys to *Tamarixia* species are available for Europe (Graham 1991), the European part of Russia and the Far East of Russia (Kostjukov 1978; Kostjukov 1995, 2000), India (Narendran 2007), North America (Burks 1943, two species as part of *Tetrastichus*), and Yemen (Yefremova and Yegorenkova 2009).

#### Key to Mexican species of *Tamarixia*

##### (Females)

**Table:** 

1	F3 subquadrate or transverse (Figs 14, 16, 19, 21), F1 1.2–1.3 times as long as F3	2
–	F3 1.8–2.0 times as long as broad (Fig. 6), F1 1.45–1.5 times as long as F3	*Tamarixia aguacatensis* sp. n.
2	Mesoscutum with complete median line	*Tamarixia radiata* (Waterston)
–	Mesoscutum with incomplete median line (Fig. 7)	3
3	Propodeum steeply inclined relative to longitudinal axis of the body	*Tamarixia schina* Zuparko
–	Propodeum inclined 45 degrees from longitudinal axis of the body (Fig. 5)	4
4	F2 as long as F3, F1 2.2 times as long as broad, clava 1.3 times as long as funicle (Fig. 16)	*Tamarixia triozae* (Burks)
–	F2 1.4 times as long as F3, F1 1.8 times as long as broad, clava 1.5 times as long as funicle (Fig. 19)	*Tamarixia leucaenae* Bouček

##### (Males)

**Table:** 

1	Pedicel 1.5 times as long as F1 (Figs 15, 17, 20, 22)	2
–	Pedicel as long as F1 or slightly longer (1.1 times as long as F1) (Fig. 8)	*Tamarixia aguacatensis* sp. n.
2	Clava 5.0 times as long as broad (Fig. 22)	*Tamarixia radiata* (Waterston)
–	Clava 4.0 times as long as broad	3
3	F2, F3 1.3–1.4 times as long as broad (Fig. 15)	*Tamarixia schina* Zuparko
–	F2, F3 1.8–2.0 times as long as broad	4
4	Whorled setae of F1 reaching the top of F3, whorls of F4 reaching top of C2 (Fig. 17)	*Tamarixia triozae* (Burks)
–	Whorled setae of F1 reaching top of F4, whorls of F4 attach out apical sensillum (Fig. 20)	*Tamarixia leucaenae* Bouček

### Description of new species

#### Tamarixia aguacatensis

Yefremova sp. n.

http://zoobank.org/2E77279C-F3E8-4C9F-97A8-4329A33AC45D

http://species-id.net/wiki/Tamarixia_aguacatensis

Figs 2 – 13

##### Holotype

(female): MEXICO, Michoacán, Salvador Escalante, Ejido El Tarascon, 19°26'29.81N, 101°49'53.03W, 1,910 m, 2.iv.2012, G. González-Santarosa (deposited in TAUI). PARATYPES (same data): 3 ♀, 3 ♂ (CNIN); 1 ♀, 1 ♂ (USNM); 2 ♀, 4 ♂ (TAUI).

##### Description.

**Figures 2–3.**
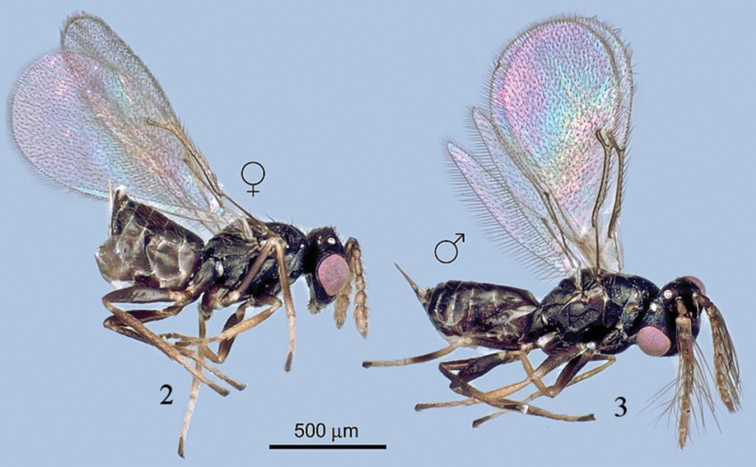
*Tamarixia aguacatensis*, female and male (habitus).

FEMALE (Fig. 2). Body length: 0.85–1.04 mm; fore wing length: 2.07–2.94 mm. Body shiny black, eye pink; antenna yellow, scape black except yellow ventrally and apically; pedicel dark dorsally and basally, yellow-brown on ventral surface; flagellar segments and clava sandy yellow; tegula yellow; legs brown dark, coxae brown, trochanters brown, trochantelli yellow, basal and distal apices of pro- and meso- femora and tibiae yellow, and metafemur and tibia brown; tarsi yellow except apical segment brown. Metanotum yellow. Gaster brown. Wings hyaline, venation brownish.

**Figures 4–9.**
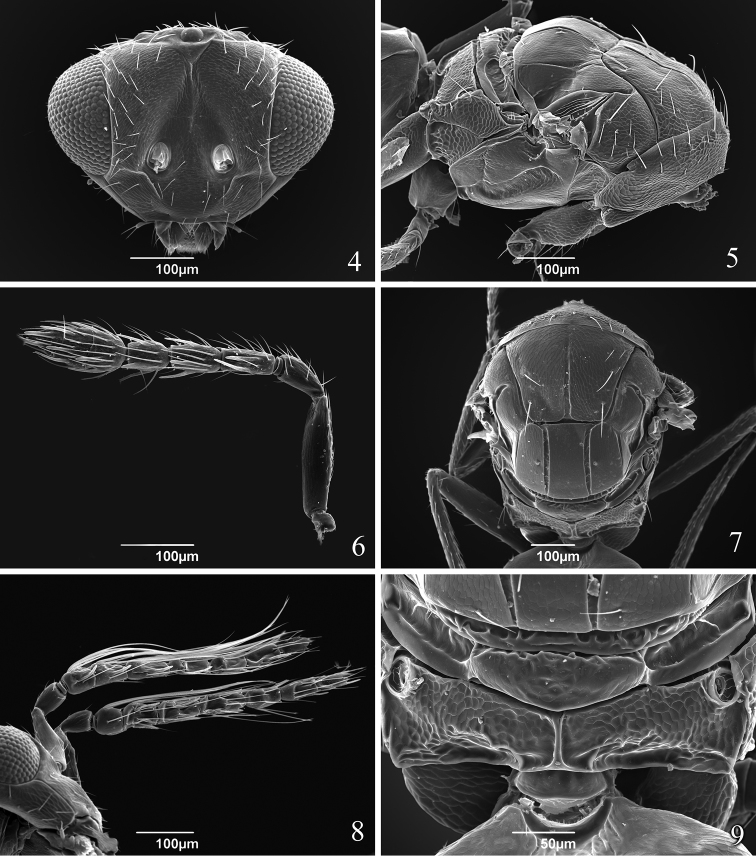
*Tamarixia aguacatensis*. Female: **4** Head, frontal view **5** Mesosoma, lateral view **6** Antenna **7** Mesosoma, dorsal view **9** Propodeum. Male **8** Both antennae on the head.

Head 2.2 times as wide as long (Fig. 4). POL 2.0–2.2 times OOL. Face smooth; vertex, frons, areas near orbits and lower face setose. Malar sulcus present. Toruli slightly above lower level of eyes. Mandible with upper long tooth and several lower short teeth. Scrobes depressed and sutured (inverted V-shaped). Eye bare. Antenna (Fig. 6) with scape 2.3 times as long as pedicel, 1 discoid anellus, pedicel as long as F1 and F2 combined, F1 2.2 times as long as broad and equal to F2, F2 2.0 times as long as broad and 1.3 times as long as F3, clava 3-segmented, 2.3–2.4 times as long as broad and 2.4–2.6 times as long as F3.

Mesosoma. Pronotum short, with 8 marginal setae (Fig. 5). Mesoscutum 1.5 times as long as broad with an incomplete median line (0.63 length of mesocutum) and with 2 pairs of long adnotaular setae (Fig. 7). Mesoscutum with additional 2 pairs of short setae in the upper part in a horizontal row and 1 seta near notauli (Figs 5, 7). Mesocutum, scutellum and dorsellum finely reticulate. Scutellum with two submedian lines closer to each other than to sublateral lines, with 2 pairs of setae; first pair of setae in the middle of scutellum. Mesosoma in lateral view higher than the plane of propodeum and inclined at an angle less than 45 degrees from the longitudinal axis of the body (Fig. 5). Propodeum (Fig. 9) strongly reticulate, with a complete simple median carina; spiracle with a rim. Callus with 2 long setae in one row (Fig. 7).

**Figures 10–13.**
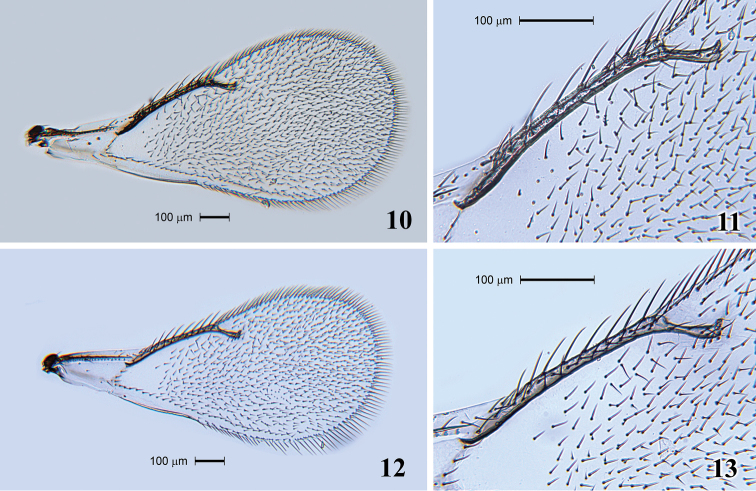
*Tamarixia aguacatensis*. Female: **10** Fore wing **11** Marginal vein with setae. Male: **12** Fore wing **13** Marginal vein with setae.

Fore wing (Fig. 10) 2.6 times as long as broad. SMV with 1 seta. Speculum extending along half length of MV and closed. SMV 1.2 times as long as MV. MV with 8 setae (Fig. 11). STV 3.4 times shorter than MV. PMV absent. Hind wing acute at apex.

Gaster 1.16–1.27 times as long as broad. Ovipositor sheaths slightly visible (Fig. 2).

MALE (Figs 3, 12, 13). Body length 0.8–1.00 mm. Colour of body very similar to that of female except gaster with tergite 1 completely yellow. Antennal scape dorsally dark brown; pedicel, and funicle sandy yellow. Coxae of all legs brown, trochanters brown, trochantelli yellow, pro- and meso- femora brown except yellow at apex, metafemur and tibia brown, tarsi yellow except apical segment dark brown. Tegula yellow. Eyes pink. Ocelli white.

Head. POL 1.6–1.8 times OOL. Antenna (Fig. 8). Scape with ventral plaque about 0.2 length in the basal half. Pedicel 1.0–1.2 times as long as F1, F2 1.1 times as long as F1, F3 1.18 times as long as F2 and equal to F4, C1 equal to C2 and C3 1.2 times as short as C2. Four funicle segments with whorled setae; whorls of F1 reaching middle of F3, whorls of F2 reaching base of F4, whorls of F3 reaching tip of C3, whorls of F4 reaching middle of C2, whorls of C1 reaching base of C3, whorls of C2 reaching middle of C3, whorls of C3 reaching apical placoid sensillum. Scutellum smooth between submedian lines, and submedian and sublateral lines. Fore wing 2.1 times as long as broad (Fig. 12). Speculum slightly larger than that in female and MV with 9 setae (Fig. 13). Metasoma. Gaster 1.65–1.8 times as long as broad. Genitalia with two long longitudinal digital sclerites. Aedeagus very long, 2.3 times as long as gaster (Fig. 3). Parameres triangular with one long parameral seta.

##### Diagnosis.

*Tamarixia aguacatensis* resembles *Tamarixia leucaenae* (examined were two female paratypes (FSCA) with the following data: Trinidad and Tobago, Trinidad Island, “UWJ Field, stn. (Lab)”, on *Leucaena* sp., det. by Z. Bouček, 1988) from which it differs by the colour of the female: legs dark brown except coxae and trochanters brown, trochantelli yellow (coxae yellowin *Tamarixia leucaenae*); in addition, the female of *Tamarixia aguacatensis* differs from that of *Tamarixia leucaenae* in having F1-F3 2.0–2.2 times as broad as long and clava 2.4 times as broad as long (F1 1.7 times as long as broad, F2 1.4 times as broad as long, F3 subquadrate and clava 2.0 times as broad as long in *Tamarixia leucaenae*).

**Figures 14–20.**
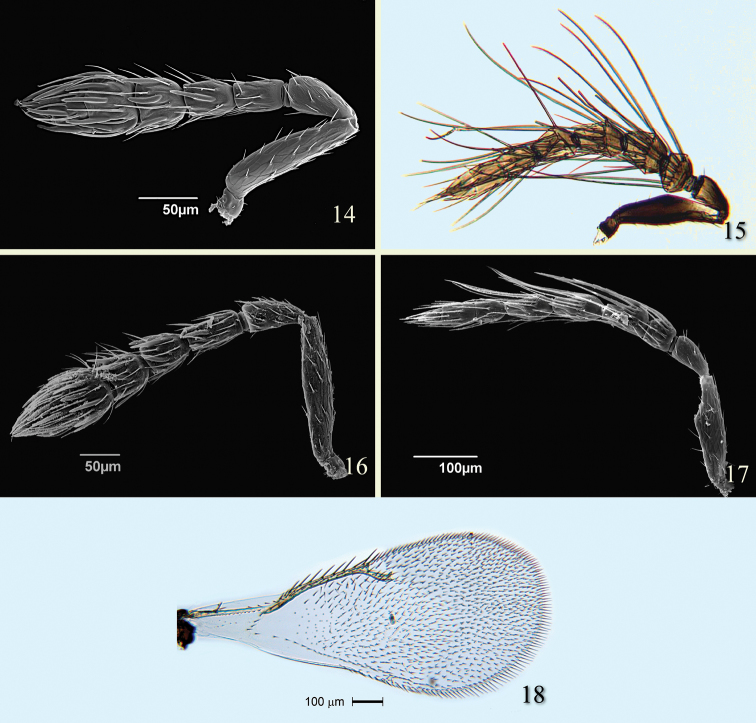
*Tamarixia schina*: **14** Female antenna **15** Male antenna. *Tamarixia triozae*: **16** Female antenna **17** Male antenna **18** Female fore wing.

The female antenna of *Tamarixia aguacatensis* differs from that of *Tamarixia schina* (Fig. 14) as follows: F1-F3 2.0–2.2 times as broad as long and clava 2.3–2.4 times as broad as long (F1 1.8 times as broad as long, F2 1.2 times as broad as long, F3 transverse, and clava 1.8 times as broad as long in *Tamarixia schina*). The male antenna of *Tamarixia aguacatensis* differs from that of *Tamarixia schina* (Fig. 15, illustrated here for the first time) as follows: pedicel equal in length to F1 (1.5 times as long as F1 in *Tamarixia schina*), F1 and F2 equal, F2 1.2 times as long asF3 (F1, F2 and F3 equal in *Tamarixia schina*), clava 2.5 as long as F3 (2.0 times as long as F3 in *Tamarixia schina*), clava 2.0 times as long as broad (1.5 times as long as broad in *Tamarixia schina*). Additionally, the metanotum and propodeum are inclined much less in *Tamarixia aguacatensis* than in *Tamarixia schina*.

Female of *Tamarixia aguacatensis* differs from that of *Tamarixia triozae* (Fig. 16) by in having F1-F3 2.0–2.2 times as broad as long and clava 2.3–2.4 times as broad as long (F1 2.0 times as broad as long, F2 1.7 times as broad as long, F3 subquadrate, and clava 1.6–1.7 times as broad as long in *Tamarixia triozae*). The male antenna of *Tamarixia aguacatensis* differs from that of *Tamarixia triozae* (Fig. 17) as follows: pedicel equal to length F1 (1.6 times as long as F1 in *Tamarixia triozae*), F1 and F2 equal to each other, F2 1.2 times longer than F3 (F1 subquadrate, F2 1.17 times shorter than F3 in *Tamarixia triozae*), clava 2.5 as long as F3 (2.2 times as long as F3 in *Tamarixia triozae*).

**Figures 19–22.**
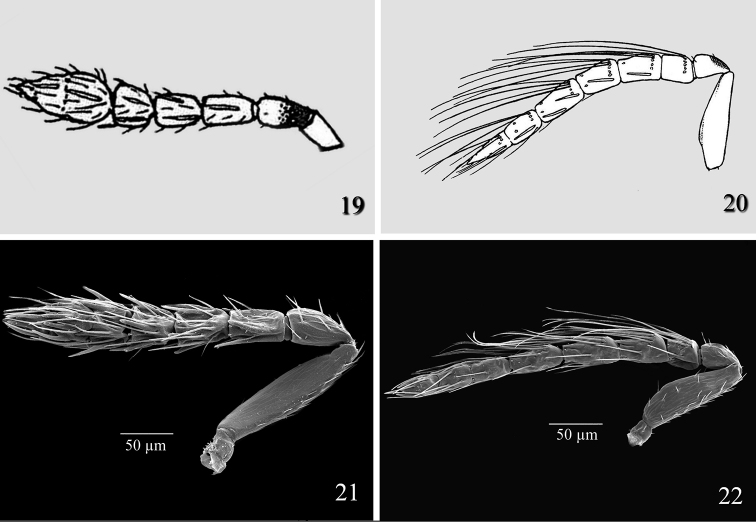
*Tamarixia leucaenae*: **19** Female antenna **20** Male antenna **21** *Tamarixia radiata*: **21** Female antenna **22** Male antenna.

Female of *Tamarixia aguacatensis* differs from that of *Tamarixia radiata* (Fig. 19) in having F1-F3 2.0–2.2 times as broad as long, clava 2.4 times as broad as long (F1 1.6 times as broad as long, F2 1.5 times as broad as long, F3 subquadrate, and clava 2.0 times as broad as long in *Tamarixia radiata*). The male antenna of *Tamarixia aguacatensis* differs from that of *Tamarixia radiata* (Fig. 20) as follows: F1 and F2 equal to each other (pedicel equal in length to F1 in both species), F2 1.2 times longer than F3 (F1 1.4 times as short as F2, F2 equal to F3 in *Tamarixia radiata*), clava 2.5 as long as F3 (5.0 times as long as F3 in *Tamarixia radiata*), whorled setae of F1 reaching middle of F3 (reaching top of F4 in *Tamarixia radiata*), whorls of F2 reaching base of F4 (Fig. 8) (reaching middle of C2 (Fig. 22) in *Tamarixia radiata*).

The male antenna of *Tamarixia aguacatensis* resembles that of *Tamarixia psyllae* Yefremova & Yegorenkova from Yemen that was reared from *Trioza erytrea* (Del Guercio) (Yefremova and Yegorenkova 2009). In the former the whorled setae of F1 reaching middle of F3 (reaching base of F2 in *Tamarixia psyllae*), whorls of F2 reaching base of F4 (reaching base of F3 in *Tamarixia psyllae*), whorls of F3 reaching top of C3 (reaching base of F4 in *Tamarixia psyllae*), whorls of F4 reaching middle of C3 (reaching C1 in *Tamarixia psyllae*).

The male antenna of *Tamarixia aguacatensis* resembles that of *Tamarixia dryi* (Waterston), reared from *Trioza citri* Laing in Kenya (Waterston 1922), from which it differs as follows: whorls of F4 reaching middle of C3 (not reaching C1 in *Tamarixia dryi*), whorls of C1 reaching base of C3 (whorls of C1 reaching 0.8 length of clava in *Tamarixia dryi*).

*Tamarixia aguacatensis* also resembles *Tamarixia flavigaster* (Brothers & Moran), described from South Africa from Psyllidae on *Calodendrum capense* (L.) (Brothers and Moran 1969), from which it differs as follows: mesoscutum with incomplete median carina, coxae brown (complete median carina and pale coxae in *Tamarixia flavigaster*), male antennal plaque about 0.2 length of scape (0.1 in *Tamarixia flavigaster*), whorled setae of F1 reaching middle of F3, whorls of F2 reaching base of F4, whorls of F3 reaching top of C3 (whorls of F1 reaching clava, whorls of F2 and F3 reaching base of C3 in *Tamarixia flavigaster*). Also, the species has a brown gaster (the gaster is almost yellow in *Tamarixia flavigaster*).

##### Distribution.

Mexico.

##### Host.

Known from *Trioza aguacate*, as a nymphal parasitoid.

##### Etymology.

The species name is derived from its host, *Trioza aguacate*.

*Tamarixia aguacatensis* is the fifth known species of *Tamarixia* in Mexico. It can be distinguished from other congeneric species in the country by having two pairs of short setae in the horizontal row on mesoscutum (Fig. 7).

## Supplementary Material

XML Treatment for Tamarixia

XML Treatment for Tamarixia aguacatensis

## References

[B1] Alvarez-Zagoya R Cibrian-Tovar D (1999) Biology of the peppertree psyllid *Calophya rubra* (Blanchard) (Homoptera: Psyllidae). Revista Chapingo, Serie Ciencia Forestales y del Ambiente 5(1): 51-57.

[B2] Bouček Z (1988a) Australasian *Chalcidoidea* (Hymenoptera). CAB International, Wallingford, UK, 832 pp.

[B3] Bouček Z (1988b) *Tamarixia leucaenae* sp. n. (Hymenoptera: Eulophidae) parasitic on the leucaena psyllid *Heteropsylla cubana* Crawford (Hemiptera) in Trinidad. Bulletin of Entomological Research 78: 545-547. doi: 10.1017/S0007485300013298

[B4] Brothers DJ Moran VC (1969) A new species of *Tetrastichus* Haliday, 1844 (Hymenoptera: Eulophidae) parasitic on the nymphs of *Paurocephala calodendri* Moran (Homoptera: Psyllidae). Proceedings of the Royal Entomological Society of London (B) 38(3/4): 40–46.

[B5] Burks BD (1943) The North American parasitic wasps of the genus *Tetrastichus* — a contribution to biological control of insect pests. Proceedings of the United States National Museum 93: 505–608. doi: 10.5479/si.00963801.93-3170.505

[B6] Day RK (1999) Integrated Control of Leucaena Psyllid. Final Technical Report of Project R6524, Funded by DFID, NR Integrational, Chayham, Kent, UK.

[B7] Gibson GAP Huber JT Woolley JB (Eds) (1997) Annotated keys to the genera of Nearctic *Chalcidoidea* (Hymenoptera). NRC Research Press, Ottawa, Ontario, 794 pp.

[B8] Graham MWR de V (1987) A reclassification of the European Tetrastichinae (Hymenoptera: Eulophidae), with a revision of certain genera. Bulletin of the British Museum (Natural History) 55: 1-392.

[B9] Graham MWR de V (1991) A reclassification of the European Tetrastichinae (Hymenoptera: Eulophidae): revision of the remaining genera. Memoirs of the American Entomological Institute 49, 322 pp.

[B10] Hollis D Martin JH (1997) Jumping plantlice (Hemiptera: Psylloidea) attacking avocado pear trees, *Persea americana*, in the New World, with a review of Lauraceae-feeding among psylloids. Bulletin of Entomological Research 87(5): 471-480. doi: 10.1017/S000748530004133X

[B11] González-Hernández A Arredondo-Bernal HC Robles-González M Martínez-Carrillo JL Pérez J López-Arroyo JI (2009) Determinación de especies de parasitoides del psílido asiático de los cítricos *Diaphorina citri* (Hemiptera: Psyllidae) en México. Entomología Mexicana 8: 373-377.

[B12] Kostjukov VV (1978) [Podsem 5. Tetrastichinae]. In: Medvedev GS (Ed) Opredelitel’ Nasekomykh Evropey’skoy Chasti SSSR, Tom III, Pereponchatokrylye, Vtoraya chast’. Nauka, Leningrad, 430-76. [in Russian]

[B13] Kostjukov VV (1990) New species of the eulophid genus *Tetrastichus* Haliday (Hymenoptera, Chalcidoidea, Eulophidae) from far eastern Russia. In: Lelei AS (Ed) Novosti sistematiki nasekomikh Dalnego Vostoka. AN SSSR, Dalnevostochnoe Otdelenie biol. pochv. in-t, Vladivostok, 46-63. [in Russian]

[B14] Kostjukov VV (1995) 46. [Family Eulophidae Subfamily Tetrastichinae]. In: Lehr PA (Ed) [Key to the insects of Russian Far East in six volumes]. 4 Dal’nauka, Vladivostok, Russia, 346-505. [in Russian]

[B15] Kostjukov VV (1996) New species of the genus *Tamarixia* Mercet (Hymenoptera, Eulophidae). Buletinul Academiei de Stiinte a Republicii Moldova. Stiinte Biologice si Chimice 4(277): 27-31. [in Russian]

[B16] Kostjukov VV (2000) [Nadsem. Chalcidoidea 46. Sem. Eulophidae]. In: Lehr PA (Ed) Opredelitel’ nasekomykh dal’nego vostoka Rossii 4(4): 582–601. Dal’nauka, Vladivostok [in Russian]

[B17] LaSalle J (1994) North American genera of Tetrastichinae (Hymenoptera: Eulophidae). Journal of Natural History 28: 109-236. doi: 10.1080/00222939400770091

[B18] León JH Sétamou M (2010) Molecular evidence suggests that populations of the Asian citrus psyllid parasitoid *Tamarixia radiata* (Hymenoptera: Eulophidae) from Texas, Florida and Mexico represent a single species. Annals of the Entomological Society of America 103: 100–120. doi: 10.1603/008.103.0113

[B19] Lomeli-Flores JR Bueno Partida R (2002) New record of *Tamarixia triozae* (Burks), parasitoid of the tomatoe [sic] psilid [sic] *Paratrioza cockerelli* (Sulc) (Homoptera: Psyllidae) in Mexico. Folia Entomológica Mexicana 41(3): 375-376.

[B20] McClay AS (1990) Distribution of leucaena psyllid and its natural enemies in Mexico: implications for biological control. Leucaena psyllid: problems and management. In: Napomopeth B MacDicken KG (Eds) Proceedings of an international workshop held in Bogor, Indonesia, January 16–21, 1989 Winrock International Institute for Agricultural Development, Bangkok 139–143.

[B21] Mead FW (1994) Eugenia psyllid, *Trioza eugeniae* Froggatt (Homoptera: Psyllidae). Entomology Circular, Florida Department of Agriculture, Gainesville, No 367: 1-3.

[B22] Mercet RG (1924) Eulófidos de España (1.a nota). Boletín de la Real Sociedad Española de Historia Natural 24: 54-59.

[B23] Moran VC Brothers DJ Case JJ (1969) Observations on the biology of *Tetrastichus flavigaster* Brothers & Moran (Hym., Eulophidae), parasitic on psyllid nymphs (Hem., Hom.). Transactions of the Royal Entomological Society of London 121: 41-58. doi: 10.1111/j.1365-2311.1969.tb00516.x

[B24] Narendran TC (2007) Indian Chalcidoid Parasitoids of the Tetrastichinae (Hymenoptera: Eulophidae). Records of the Zoological Survey of India, Occasional Paper No. 272, 1–386 + 5 plates.

[B25] Noyes JS (2013) Universal Chalcidoidea Database, World Wide Web electronic publication. The Natural History Museum, London http://www.nhm.ac.uk/entomology/chalcidoids/index.html [accessed on 23 August 2013]

[B26] Patil NG Baker PS Pollard GV (1993) Life histories of *Psyllaephagus yaseeni* (Hym., Encyrtidae) and *Tamarixia leucaenae* (Hym., Eulophidae), parasitoids of the leucaena psyllid *Heteropsylla cubana*. Entomophaga 38: 565-577. doi: 10.1007/BF02373091

[B27] Pluke RWH Qureshi JA Stansly PA (2008) Citrus flushing patterns, *Diaphorina citri* populations and parasitism by *Tamarixia radiata* in Puerto Rico. Florida Entomologist 91: 36-42. doi: 10.1653/0015-4040(2008)091[0036:CFPDCH]2.0.CO;2

[B28] Rao MR Singh MP Day R (2000) Insect pest problems in tropical agroforestry systems: Contributory factors and strategies for management. Agroforestry Systems 50: 243-277. doi: 10.1023/A:1006421701772

[B29] Schauff ME LaSalle J Coote LD (1997) Chapter 10. Eulophidae. In: Gibson GAP Huber JT Woolley JB (Eds) Annotated keys to the genera of Nearctic *Chalcidoidea* (Hymenoptera). NRC Research Press, Ottawa, Ontario, 327-429.

[B30] Waterston J (1922) On the chalcid parasites of psyllids (Homoptera). Bulletin of Entomological Research 13(1): 41-58. doi: 10.1017/S0007485300045235

[B31] Yefremova ZA Yegorenkova EN (2009) The subfamily of Tetrastichinae (Hymenoptera: Eulophidae) in Yemen, with description of new species. Fauna of Arabia: 169–211.

[B32] Zuparko RL De Queiroz DL LaSalle J (2011) Two new species of *Tamarixia* (Hymenoptera: Eulophidae) from Chile and Australia, established as biological control agents of invasive psyllids (Hemiptera: Calophyidae, Triozidae) in California. Zootaxa 2921: 13-27.

